# DCBRP: a deterministic chain-based routing protocol for wireless sensor networks

**DOI:** 10.1186/s40064-016-3704-1

**Published:** 2016-11-29

**Authors:** Haydar Abdulameer Marhoon, M. Mahmuddin, Shahrudin Awang Nor

**Affiliations:** 1 College of Science, Computer Department, University of Karbala, Kerbala, Iraq; 2 InterNetWorks Research Lab, School of Computing, College of Arts and Sciences, Universiti Utara Malaysia, Sintok, Kedah Malaysia

**Keywords:** Chain head selection, Deterministic chain-based routing protocol, Wireless sensor network

## Abstract

**Background:**

Wireless sensor networks (WSNs) are a promising area for both researchers and industry because of their various applications The sensor node expends the majority of its energy on communication with other nodes. Therefore, the routing protocol plays an important role in delivering network data while minimizing energy consumption as much as possible. The chain-based routing approach is superior to other approaches. However, chain-based routing protocols still expend substantial energy in the Chain Head (CH) node. In addition, these protocols also have the bottleneck issues.

**Methods:**

A novel routing protocol which is Deterministic Chain-Based Routing Protocol (DCBRP). DCBRP consists of three mechanisms: Backbone Construction Mechanism, Chain Head Selection (CHS), and the Next Hop Connection Mechanism. The CHS mechanism is presented in detail, and it is evaluated through comparison with the CCM and TSCP using an ns-3 simulator.

**Results:**

It show that DCBRP outperforms both CCM and TSCP in terms of end-to-end delay by 19.3 and 65%, respectively, CH energy consumption by 18.3 and 23.0%, respectively, overall energy consumption by 23.7 and 31.4%, respectively, network lifetime by 22 and 38%, respectively, and the energy*delay metric by 44.85 and 77.54%, respectively.

**Conclusion:**

DCBRP can be used in any deterministic node deployment applications, such as smart cities or smart agriculture, to reduce energy depletion and prolong the lifetimes of WSNs.

## Background

 A WSN refers to a large number of sensor nodes that are connected to one another. WSNs are widely applied in various areas, such as the military, industries, environment, disaster management, and habitat monitoring (Sikander 2013). All sensor nodes have limitations, for example, in bandwidth, computational ability, power resources, and memory (Gautam et al. 2009). These nodes have the ability to communicate with one another wirelessly with one or more unlimited-energy resource nodes, called Base Stations (BSs), which may be connected to the Internet. A sensor node consists of four elements: the first sensor collects specific data from the environment; the second is the radio module, which is responsible for sending and receiving data via a wireless medium; the third is the micro controller for processing purposes; and the fourth is the power supply, which provides the necessary power to all sensor-node components in the device (Hadjila et al. 2013). Typically, the main power source is the battery. However, due to its deployment strategies, recharging is an impossible task. Therefore, WSN nodes have a certain level of algorithmic intelligence to collect data and send them to the BS due to energy considerations (Wei et al. 2011).

Sending the network packets is a critical challenge that directly affects the performance of sensor nodes. The main purpose of developing a new routing protocol in WSNs is to reduce the energy consumption and extend the network lifetime of the sensor nodes. The performance of a WSN can also be affected by other factors, such as bandwidth, scalability, data aggregation, energy consumption, mobility, multipath, redundancy, end-to-end delay, packet loss, network load, and localization (Shukla et al. 2014).

Cluster-based, chain-based, and tree-based protocols are the basic classifications of the hierarchical routing protocols (Zhang et al. 2010). While under cluster-based protocols, some nodes are selected to be the cluster heads, and other nodes are connected to the closest cluster heads as normal nodes. A good example of this is the Low-Energy Adaptive Clustering Hierarchy (LEACH), along with its family of protocols (Heinzelman et al. 2000). In the LEACH protocol, the normal nodes sense the environment and send the data to their cluster heads using a single-hop method. Subsequently, the cluster heads will also deliver the network data to the BS in a single-hop manner. Therefore, energy consumption can be considered a significant problem in this approach because in most cases, the single hop will involve long-distance communication. The principal concept in Tree-based routing is data transmission only from children (sensor nodes) to their parent (Liang et al. 2009). An example of a Tree-based routing protocol is the DRINA routing protocol (Villas et al. 2013). However, the main issue with DRINA is that it suffers from high energy consumption in nodes over the network lifetime. The Chain-based approach is more promising than the other approaches with respect to connection behaviour in power conservation (Mamun et al. 2010; Mamun 2012). Within the Chain-based approach, every node is connected with its neighbours to reduce the consumption of energy caused by long-distance communication among nodes. Nevertheless, like many other approaches, the Chain-based approach is not perfect. Chain-based routing protocols still have drawbacks, especially in the single long chain (Marhoon et al. 2015). This paper presents the critical issues in WSN routing protocols and (specifically) intends to find the most appropriate chain head, and determine the optimal number of chain heads in a network.

## Theoretical analysis of chain head selection

The chain head selection is a high-priority phase in most WSN routing protocols. It is an essential step in the chain-based approach, as all protocols have a similar mechanism in some respects. A normal sensor node transmits its own data to the nearest node in the same chain and therefore expends little energy compared to the chain head. It is important to realize that the chain head is responsible for transferring all data (related to the chain or network) to the BS. Therefore, it requires substantial energy to ensure that all data are transferred without any packet loss.

Furthermore, the method used to select the proper node to be the chain head is important for prolonging the network lifetime and keeping all sensor nodes connected. The number of chain heads can directly affect the performance of a protocol by dividing the responsibility of data delivery to the BS and dividing the required energy over a number of chain heads. This is not only for the purpose of reducing energy consumption but also for reducing delays that are caused by the single network gateway.

Chain Routing Based on Coordinates-oriented Cluster (CRBCC), proposed in Gengsheng et al. (2009), selects the chain head on the basis of node position on top of the chain. In addition, the CRBCC protocol selects the main head randomly, which is considered the main drawback of this protocol. The Balanced Chain-Based Routing Protocol (BCBRP) (Ahn et al. 2011) also selects the main head randomly after dividing the network into small subnetworks. The Energy-Efficient Chain-Based Routing Protocol (EECB) (Yu and Song 2010) is an improved version of the PEGASIS protocol. It uses Eq. 1 to select the chain head node.

1 $$Q_{i} = E_{res - i} /d_{i} ,$$

where *Q*_*i*_ is the comparative factor, *E*_*res*_ is the residual energy of the nodes, and *d*_*i*_ is the distance between the nodes and the BS. Furthermore, the Rotation PEGASIS-Based Routing Protocol (RPB) (Yang et al. 2013) selects its chain head on the basis of Eq. 2, which depends on the residual energy and distance factors in addition to two weight parameters. These parameters include the distance and energy, and can be used to manipulate the important factor based on the requirements.

2 $$Q_{i} = W_{1} * E_{i} + W_{2} /d_{BS\left( i \right)} ,$$

where *W*_*1*_ and *W*_*2*_ are the weight parameters. The Energy-Efficient Cluster-Chain Based Routing Protocol (ECCP) (Sheikhpour and Jabbehdari 2012) determines the weights according to the number of node neighbours, and selects the node that has maximum Wi according to Eq. 3, where Wi is a comparative variable.

3 $$W_{i} = RE_{i} * \sum\limits_{j = 1}^{no.\;of\;neighbor} {1/d^{2} \left( {v_{i} ,v_{j} } \right)}$$

In addition, the Improved Energy-Efficient PEGASIS-Based (IEEPB) routing protocol, proposed in Feng et al. (2011), selects in the second phase the chain head on the basis of Eqs. 4, 5, and 6 to satisfy the balance between energy and distance when the chain head node is chosen.

4 $$D_{bs} = d_{BS}^{4} /d_{ave}^{4}$$

5 $$E_{p} = E_{inti} /E_{rem}$$

6 $$W_{i} = w_{1} * E_{p} + w_{2} * D_{bs}$$

Based on the discussion in the literature on how to select the chain heads in the Chain-based routing protocols, there are many ways to assign this role to the correct node. For example, the PEGASIS protocol is used at random to select the chain head to ensure that the first dead node is located at a random position in the sensing area. Moreover, the rotating selection of the same node after *i* rounds for *N* nodes is achieved by applying Eq. 7.

7 $$CH = i\;\bmod \;N$$

CCM (Tang et al. 2010) and TSCP (Kareem et al. 2014) use the sequence method for chain head selection (ignoring the amount of energy expended by the nodes) and the remaining energy to select the main head (connected with the BS) and ignore the node position relative to the BS, as expressed in Eq. 8.

8 $$S_{factor} = E_{remaining} ,$$

where *E*_*remaining*_ refers to the remaining energy in the sensing node and *S*_*factor*_ is the selection factor for choosing the main head.

In contrast, the most appropriate method for chain head selection (as in some other routing protocols) is dependent on the residual energy divided by the distance from the BS, because of the consideration of node specifications rather than randomization. However, it does not consider the data delivery ability of the nodes. Consequently, the CHS mechanism uses a proactive selection mechanism to manage the relevant residual energy and the ability of the node for data delivery.

The Chain-Cluster Mixed-Routing protocol (CCM) attempts to combine the strong points of the cluster and chain approaches through the following actions:

1. Connecting all nodes in the same row as a chain: This means that the CCM has ten horizontal chains for a network with ten rows. This is beneficial, as it will help to reduce the power consumption;
2. Choosing the chains heads using the Sequence Method, which ignores node ability. It chooses the main head according to only the remaining energy; and
3. Depending on the distance factor, it chooses the next hop connections over the entire network lifetime, while using a cluster approach to choose the next hop connections between the chains heads and the main head to reduce delay.

The Two-Stage Chain-Based routing protocol (TSCP) applies the chain approach completely for both intra- and inter-connections to take advantage of the chain concept to reduce the energy consumption. Therefore, it makes improvements in chain head connections and uses the following operations to route the sensing data:

1. Connecting all nodes in the same row as a chain, which is the same method used in the CCM protocol;
2. Choosing the chains heads using the sequence method in the early rounds, and then when the nodes deplete most of the energy, these chains heads will be selected according to only the remaining energy; and
3. The next hop connections are chosen based on only the distance factors in the earlier rounds; then it will ignore all other factors to make connections between chain heads.

TSCP takes full advantage of the chain concept, but there is a trade-off with the delay metrics. It will force many packets to pass to the main head through unnecessary nodes, which will result in unnecessary energy consumption. Furthermore, chain heads are connected to each other in chain form to reduce the power consumption; however, this will increase the hop counts for all packets that travel from the source to the destination.

## Significance and problem statement

The energy required to transmit one bit is equal to the energy required to execute 3000 processes (Eslaminejad and Razak 2012). The communication part is then considered the main source of energy consumption in the sensor node. Choice of routing protocol is one of the most important issues directly influencing the performance of the WSN in the communication part. The main goal of routing protocols in WSNs is to deliver the sensing data to the BS with minimum power consumption and maximum lifetime. The chain-based routing protocol performs this task with minimum power consumption and prolongs the network lifetime (Mamun 2012; Kareem et al. 2014). Furthermore, the chain-based approach confidently reduces communication energy consumption using low radio power in order to connect each node to its closest neighbour (Liang et al. 2009).

However, this chain is subject to failure in the chain head when all network data are sent to the single leader node. This node is responsible for delivering all network data to the BS (Liu 2015). The main node expends its energy rapidly, and its consumption is too high compared to other nodes. Therefore, the main node needs to be selected efficiently because a single leader can also cause bottleneck issues in the network (Sikander 2013; Rahman et al. 2013; Taghikhaki et al. 2013).

## Research methodology

In this research, the steps are applied systematically from the first stage until the research findings are obtained. Reliable resources are very important to elicit and attain a high degree of confidence in the output. Therefore, the first step is to address powerful search engines, such as Web of Science (WoS), SCOPUS and Google Scholar. In addition, trustworthy databases, such as Science Direct, IEEEXplore, SpringerLink, and the ACM digital library offer a comprehensive literature review related to WSN routing protocols.

**Fig. 1:**
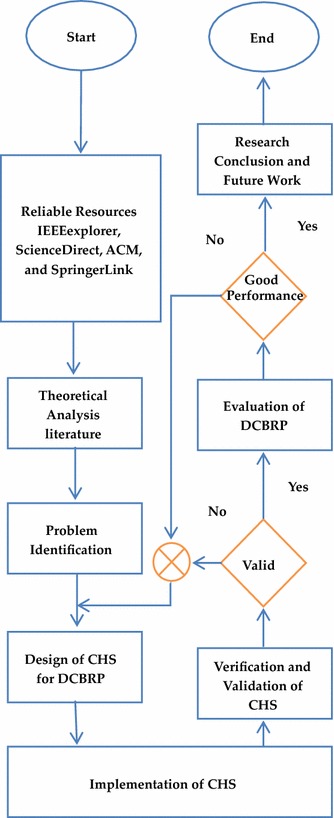
The research methodology steps for the CHS mechanism

The next step is to identify the problem statement, which focuses on how to select the most efficient and reliable chain head for data delivery. Subsequently, the proposed mechanism is designed, implemented, verified, validated, and evaluated using some of the most popular WSN metrics, such as energy consumption and network lifetime. Figure 1 illustrates the logical sequence of the steps used in this research.

Moreover, CHS mechanism design stars from the mathematical model to calculate the CHS_factor_ which is used for nodes selection. Ns-3 used in implementation part, therefore C++ is required for simulation scenario. While the Eclipse environment will be helpful for verification part, furthermore mathematical model will validate by take the real data from the simulation to confirm that CHS Equation was implementation correctly. The evaluation part is very important to ensure that DCBRP can overcome the other protocols in terms of the most important WSN metrics.

## A deterministic chain-based routing protocol (DCBRP)

**Fig. 2:**
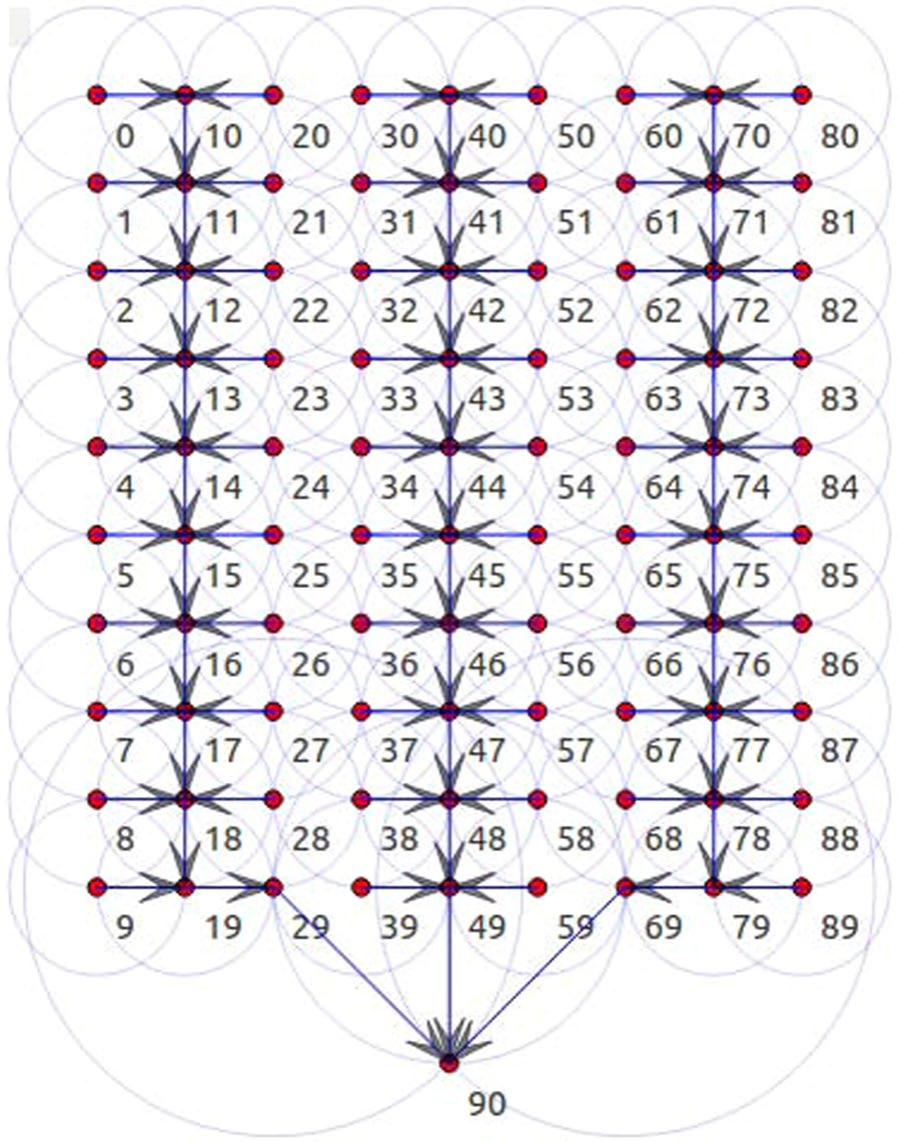
Packets forwarding according to DCBRP
- *Chain Head Selection Mechanism (CHS):* This is the second mechanism in the DCBRP. It is responsible for choosing the number of chain heads in the network, which are connected directly with the BS in each cluster. Furthermore, the CHS mechanism is very important for reducing the power consumption of chain head nodes and prolonging the network lifetime, particularly when the first node dies (FND). This paper focuses on this mechanism in detail.
- *Next*-*Hop Connection Mechanism (NHC):* The NHC mechanism is the third part of DCBRP. Its main goal is to choose the proper next hop for data delivery for each level to prevent using a weak (low-energy) node in the main chain and to avoid link failure during data collection. This is accomplished using Eq. 11.
  11 $$NHC_{factor} = \frac{{E_{Initial} - \mathop \sum \nolimits_{0}^{current\, round} E_{consumption} }}{{\sqrt[2]{{\left( {Y_{B} - Y_{A} } \right)^{2} + \left( {X_{B} - X_{A} } \right)^{2} }}}},$$
  where NHC_factor_ is a comparison factor for choosing the NHC, E_initial_ is the initial energy of the nodes, E_consumption_ is the energy consumption by the nodes, and Y and X are the node positions. Every round, nodes sends their energy to BS and BS calculates the NHC_factor_ and assign NHC for every row and so on.

The main purpose of this research is to establish an energy-efficient routing protocol for WSNs to prolong network lifetime and reduce power consumption. The proposed protocol is designed for the deterministic deployment of sensor nodes. It consists of three main mechanisms:

- *Backbone Construction Mechanism (BCM):* This mechanism is responsible for computing the number of clusters in the network and calculating the number of columns in each cluster using Eqs. 9 and 10. The main goal of BCM is to reduce the long chains and is therefore considered the main mechanism for delay minimization.
  9 $$N_{cluster} = \left\lceil {\frac{{N_{columns} }}{3}} \right\rceil$$
  10 $$N_{cluster} \;\bmod \, 3 = \left\{ {\begin{array}{*{20}l} {1, \;then\, c_{n - 1} , \;c_{n} = 2, 2} \hfill \\ {2, \;then\, c_{n - 1} , \;c_{n} = 3, 2} \hfill \\ {0, \;then\, c_{n - 1} ,\; c_{n} = 3, 3} \hfill \\ \end{array} } \right.$$
  *N*_*cluster*_ is the number of clusters in the network and *N*_*columns*_ is the number of columns in the network. The network is divided into clusters based on Eq. 9 and each cluster has a number of columns, calculated using Eq. 10. BCM is important for describing the node connectivity in the DCBRP, as shown in Fig. 2.

In comparison with other mechanisms, the DCBRP can route the network data from the source to the destination along a reliable path, with significantly less power consumption, less delay, and increased network lifetime. These metrics are considered extremely important in the performance evaluation of the WSN. Therefore, in the very first round of the network, the DCBRP has some assumptions as follows:

- The BS has global knowledge about the number of nodes’ columns and the number of rows as well as the total number of nodes in the network.
- All nodes are homogenous and they can play the same role in the network, which are sensing, relay the previous data or chain head.
- All nodes have adjustable radio power signals to make sure they can connect with close neighbor only with low power consumption and apply the chain approach concept.
- All nodes and base station have fixed position (stationary).
- Symmetric channel used in this research means the needed energy for transmission from A to B is the same as the required energy from B to A.
- For nodes deployment in the sensing area, deterministic deployment way is used to distribute the sensor nodes with equal distance between them.

## The design of the chain head selection mechanism (CHS)

The second phase of the DCBRP is the CHS mechanism. This mechanism is responsible for selecting one chain head in each cluster in the sensor network. Therefore, there are *N* chain heads in a network that has *N* clusters. Initially, the BS will calculate the number of chain heads, and then it will try to find the minimum value of the selection factor (CHS_factor_). CHS_factor_ is a factor that is calculated by dividing the amount of power consumption of this node by the remaining energy of the same node. In the CHS mechanism, the BS needs to perform the following tasks:

- Receive the remaining energy of all network nodes,
- Calculate the CHS_factor_ for every node in the cluster,
- Compare CHS_factor_ values of all nodes and choose the minimum value, and
- Broadcast the chain-head selection decisions to all sensing nodes.

In the first step and at the end of each round, each node sends its remaining energy to the BS to start the CHS_factor_ calculation for all nodes based on their own characteristics and abilities for data delivery. The BS will make a comparison to obtain the minimum value of CHS_factor_ for all nodes in the same cluster and select the CH nodes for each cluster in the network. Finally, the BS will broadcast the CHS mechanism’s decisions to the nodes in all clusters and wait for the next phase.

The main idea behind the CHS mechanism is the method for calculating the CHS_factor_. This method involves applying the CHS mechanism to choose the chain node that expends the minimum amount of energy from its remaining energy to transmit data to the BS.

The power consumption for *k* bits and distance *d* is

12 $${\text{E}}_{\text{consumption}} = {\text{E}}_{\text{elec}} * {\text{k}} + {\text{E}}_{\text{amp}} * {\text{k}} * d^{2} ,$$

and E_remaining_ is the remaining energy in every node, so

13 $${\text{CHS}}_{\text{factor}} = \frac{{{\text{E}}_{\text{consumption}} }}{{{\text{E}}_{\text{remaining}} }}.$$

This is obtained from the CHS mechanism requirements, and

14 $${\text{E}}_{\text{remain}} = {\text{E}}_{\text{Initial}} - \mathop \sum \limits_{0}^{{{\text{current}}\;{\text{round}}}} {\text{E}}_{\text{consumption}} .$$

Therefore, from Eqs. (12), (13) and (14),

15 $${\text{CHS}}_{\text{factor}} = \frac{{{\text{E}}_{\text{elec}} * {\text{k}} + {\text{E}}_{\text{amp}} * {\text{k}} * {\text{d}}^{2} }}{{{\text{E}}_{Initial} - \mathop \sum \nolimits_{0}^{{{current}\;{round}}} {\text{E}}_{consum} }},$$

where *E*_*elec*_ is the amount of energy expended to open the electronic circuit, E_amp_ is the amount of energy expended by the amplifiers in the sensor nodes, d is the distance between the node and the BS, and *E*_*initial*_ is the initial energy of the node.

By applying Eq. 15, the CHS mechanism is considered a proactive mechanism because it measures the data delivery abilities before the selection of nodes. As a result, the DCBRP will not lose any data during the CH transmission phase because it has already selected the strongest node as the chain head, as demonstrated in the above Equation.

Figure 2 shows that the CHS mechanism selects three chain heads for three clusters, depending on the number of clusters in the sensor network.

## Energy consumption model

The First-order Radio Model is used in this research as the energy model and is also employed in most routing protocols in WSN, such as Heinzelman et al. (2000), Lindsey and Raghavendra (2002), Xu et al. (2015), Sumithra and Victoire (2014), Singh et al. (2016) and Liu (2015). In this model, the required energy for the running, receiving, and transmitting circuit is E_elec_ = 50 nJ/bit. In addition, the required energy for the transmitting amplifier is E_amp_ = 100 pJ/bit/m2. Therefore, Eq. 13 is used to transmit *k* bits of data from one node to other nodes over distance *d*. Equation 14 is used to receive *k* bits at the destination node.

**For** ***k*** **-** ***bit*** **transmission**

$$E_{TX} \left( {k,d} \right) = E_{TX - elec\left( k \right)} + E_{TX - amp} \left( {k,d} \right),$$

16 $$E_{TX} \left( {k,d} \right) = E_{elec} * k + E_{amp} * k * d^{2} ,$$

**Fig. 3:**
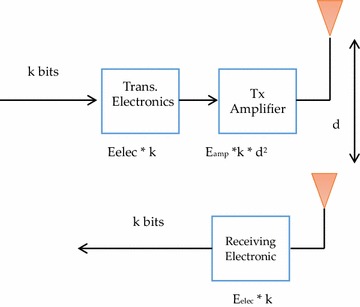
First-order radio model

**For** ***k*** **-** ***bit*** **receiving**

$$E_{Rx} \left( k \right) = E_{Rx - elec} \left( k \right),$$

17 $$E_{Rx} \left( k \right) = E_{elec} * k,$$

where E_TX_ is the transmission energy, E_elec_ is energy needed to run the transmitting circuit for 1 bit, E_amp_ is the energy required for the amplifier for 1 bit of m^2^, *k* is the number of bits and E_Rx_ is the energy required to receive *k* bits. Figure 3 shows the basic elements of the first-order radio model (Heinzelman 2000).

This research makes the same assumption as in Heinzelman et al. (2000) and Lindsey and Raghavendra (2002), that the radio channel in the network is symmetric. This means that the energy required for sending a packet from node A to node B is the same as that required to send the same packet from node B to node A.

## The implementation of the CHS mechanism

**Fig. 4:**
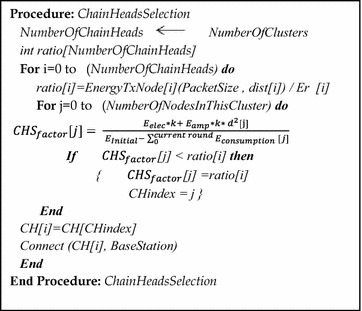
Pseudo code for the CHS mechanism of the DCBRP

The Network Simulator 3 (ns-3) (Henderson et al. 2006) is used to implement the Chain-Head Selection mechanism, i.e., for the implementation of Eq. 12, which pertains to the connection management of the chain heads with the BS. The early chain head determines the distance between each individual node and the BS. This can be measured using the signal strength or the distance function in the Static Grid Mobility model, which is included in ns-3. Figure 4 explains the pseudo code of the CHS mechanism and its steps.

To determine the number of chain heads in the network, the CHS mechanism receives the number of clusters from the previous mechanism (BCM). In addition, the mechanism works to assign one CH to every cluster, which will be responsible for transferring all data in this cluster to the BS during this round. After that, the ns-3 Mobility model will calculate the distances between the nodes and the BS. Additionally, the TX energy consumption needs to be calculated by calling the transmitting energy consumption function. The BS first requires the packet size, distance, and remaining energy to obtain the CHS_factor_ and then selects the nodes that have minimum values of CHS_factor_ as the chain heads.

## Verification and validation

Verification is an important step to confirm that the CHS has been correctly converted from the pseudo code to the programing language. The Eclipse IDE C++ is used to ensure that the CHS mechanism is free from errors after it is programmed in C++ (as the ns-3 requires). The validation of the CHS consists of two parts: The first is to examine Eq. 12 to ensure that it will provide the same expected results using both the mathematical and simulation approaches. It then tests its behaviour in the ns-3 simulator by investigating the energy consumption in different rounds, for instance, 100, 250, 500, and 700. Furthermore, it tests the CHS mechanism when it is applied in the DCBRP. Finally, it is compared with the Direct-connection method (in which no chain head is required Lindsey and Raghavendra 2002) and the BS.

### Validation of CHS_factor_ equation

CHS_factor_ is considered the main element in this mechanism. Table 1 presents five cases for CHS mechanism operations in ns-3 to calculate the CHS_factor_ and to obtain the chain head node in the first cluster (30 nodes). The chain head selections are based on the factors, presented in Table 1 and used in Eq. 12. It means that Node28 is more suitable node in round 100 to play the role of chain head because it spends little energy from its remaining energy to deliver the network data to the BS.

**Table 1:** CHS_factor_ obtained from ns-3 for different rounds

Rnd no.	Remaining energy	Distance with BS	Energy consumption for 1 Packet	CHS_factor_	Node ID (CH)
100	1.65056	36.0555	0.00036864	0.000223	Node28
250	1.3015	44.7214	0.000512001	0.000393393	Node27
500	0.829764	58.3095	0.00079872	0.000963	Node16
750	0.367199	63.2456	0.000921601	0.002509814	Node25
900	0.199025	117.047	0.00290816	0.014612034	Node0

Furthermore, mathematical calculation is required in this Section to confirm the obtained results from the ns-3 simulation. The data captured from ns-3 simulator for rounds 100, 250, 500, 750, and 900 are used to obtain the CHS_factor_ using the following calculations:

Case round 100:

$$\begin{array}{*{20}l} {{\text{Remaining}} = 1.65056;\quad {\text{E}}_{\text{elec}} = 50 * 10^{-9}\; {\text{and}}} \hfill \\ {{\text{E}}_{\text{amp}} = 100 * 10^{-12};\;k = 2048;\quad {\text{distance}}\,\left({28,BS} \right) = 36.0555} \hfill \\ \end{array}$$

$$\begin{aligned} {\text{CHS}}_{\text{factor}} = &\, \frac{{{\text{E}}_{\text{elec}} * {\text{k}} + {\text{E}}_{\text{amp}} * {\text{k}} * {\text{d}}^{2} }}{{{\text{E}}_{\text{Initial}} - \mathop \sum \nolimits_{0}^{{{\text{current}}\;{\text{round}}}} {\text{E}}_{\text{consumption}} }} = \frac{{ 50 * 10^{ - 9} * 2048 + 100^{ - 12} * 2048 * 36.0555^{2} }}{ 1.65056} \\ {\text{CHS}}_{\text{factor}} &= 0.000223; \\ \end{aligned}$$

and this is the minimum CHS_factor_ in round number 100, which is stacked with Node28. This node will play the role of chain head for this round. This calculation needs to be repeated for round number 250, 500, 750, and 900, respectively. Therefore, it attains the value of CHS_factor_ and shows that it is the same value, as presented in Table 1.

### Validation of CHS mechanism with the direct method

In the direct method, all nodes send their data directly to the BS without any mechanism for choosing the chain head, and thus all nodes will suffer from long-distance communication with the BS. The distance is an important parameter in the Equation of the energy consumption. Thus, the energy consumption is examined using the CHS mechanism to compare the protocol with a protocol that does not have it. This comparison is based on the energy consumption in the first round of the WSN simulation environment.

**Fig. 5:**
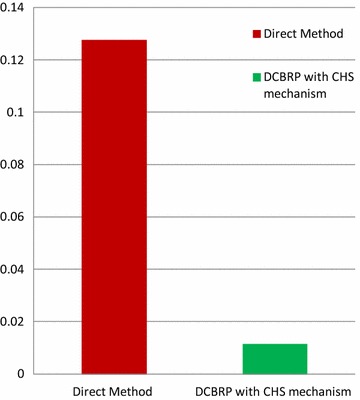
Energy consumption in the 1st round by all nodes

Figure 5 shows that the energy consumption of all nodes in the direct method for the 1st round is 0.127488. However, it is 0.0114074 with the CHS mechanism in the DCBRP. This experiment confirms two things:

- The CHS mechanism has been successfully applied in the WSN environment, and
- The CHS mechanism can reduce energy consumption significantly.

Moreover, a sensor node needs to send its data to the chain head only by using a multi-hop method in the cluster’s chain. The chain head will forward the data to the BS in a single-hop fashion. Therefore, this mechanism works to save the energy of all network nodes to prolong the network lifetime (i.e., extend the sensing rounds) and reduce the power consumption.

## Performance evaluation of DCBRP

This research uses a deterministic sensor-node deployment in a sensing-network area. The parameters and settings presented in Table 2 are required to evaluate the DCBRP and its three proposed mechanisms (BCM, CHS, and NHC). These settings are used because they have been considered by several researchers, such as in Feng et al. (2011), Tang et al. (2010), Kareem et al. (2014), Lindsey and Raghavendra (2002), Singh et al. (2016), Ali and Refaay (2011), Shiva et al. (2014) and Ganesh and Amutha (2013). Moreover, the DCBRP is compared with the most similar routing protocols in the same determinist node deployment using popular WSN performance metrics.

**Table 2:** Simulator parameters

Parameter	Value in scenario 1
Sensing area	100 * 100 m
Topology	Grid of size 9 × 10
Number of nodes	90
BS location	(50,120)
Initial node energy	2.0 J
Packet length	2 Kbit
Node deployment	Deterministic
Distance between nodes	10 m
Energy consumption model	First-order Radio Model
Energy spent to send/receive	50 nJ/bit
Routing protocol	DCBRP, TSCP, CCM

The key step in all performance evaluations is the selection of performance metrics (Al-Momani 2010). Here, we compare the performance of the DCBRP with those of the CCM and TSCP. Selecting the proper performance metrics is very important for investigating the behaviours of the protocol from different perspectives. The use of multiple different metrics gives us a complete view of the performance of the proposed mechanism.

### Delay

Delay is considered the main drawback of the Chain-based routing protocols. Thus, it is important to enhance the delay criteria when designing the primary temporal-evaluation metric. In the literature, this evaluation metric is divided into two sub-metrics.

**Fig. 6:**
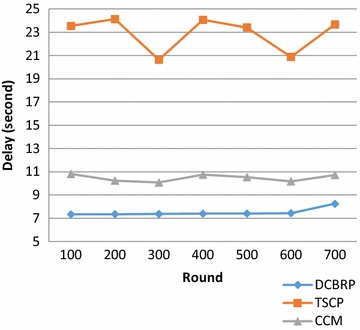
Average end-to-end delay for DCBRP, TSCP and CCM
*Number of chains in the network*: This comes from the first mechanism in the protocol (for the DCBRP, it comes from the BCM mechanism).
*Long Chains*: This also comes from the first mechanism.
*Number of chain heads*: This can be computed from the second mechanism, i.e., the CHS mechanism.
*Chain*-*heads connections*: These are the connections through which the CHs will communicate with one another.

A. Average delay every 100 rounds: this can be calculated by dividing the sum of the end-to-end delays for all packets by the total number of packets in this round, as shown in Eq. 18.
  18 $$AveDelay = \mathop \sum \limits_{P = 0}^{Last\;p} Trx - Ttx/NumberOfPackets$$
B. Average end-to-end delay for the network lifetime: this metric measures the overall delay over the protocol lifetime, as shown in Eq. 21.
  19 $$OverAllDelay = \mathop \sum \limits_{round = 0}^{last round} AvergDelay/LastNodeDieRound$$
  Figure 6 shows that the DCBRP outperforms both the CCM and TSCP. The delay metric can be affected by many things:

From the above points, the DCBRP has a lower delay than the Chain-based approach, in which each node is connected with its neighbours only. Additionally, the DCBRP reduces the total number of chains and the number of long chains by applying the BCM mechanism, which selects a specific number of clusters (backbone chains) according to its Equations. The CCM and TSCP apply the same method when assigning one chain for every row in the network. The delay also depends on the width of the network, as multi-hops are constructed along chains, increasing the travelling time for the packets from the source to the BS.

**Fig. 7:**
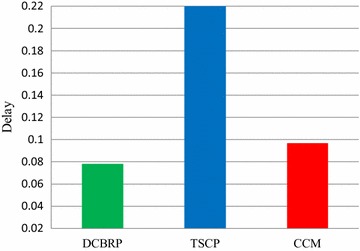
Average end-to-end delay until LND Rounds for DCBRP, TSCP and CCM

Figure 7 illustrates the average end-to-end delay until LND and the average end-to-end delay for the entire network lifetime. Since the delay metric will measure the average end-to-end delay until the FND, this metric measures the total average end-to-end delay until all nodes die and thus it will give a good view from which to evaluate the performance of these protocols. Even though some of the nodes are lost, other nodes still have packets to be transmitted to the BS.

### Network lifetime

The main performance metric in WSNs is the network lifetime due to the constraint on power resources in all nodes (all nodes depend on battery as the power supply) (Mamun 2012). The network lifetime is considered a critical issue for researchers when they design a WSN routing protocol. In this research, it was considered very important to measure the network lifetime according to the following criteria: (a) The round at which the first node dies (Chen and Zhao 2005; Kang and Poovendran 2005; Shi et al. 2006); (b) The round at which 50% of the nodes die (Raicu et al. 2005; Perillo and Heinzelman 2008) and (c) The round at which all nodes die.

**Fig. 8:**
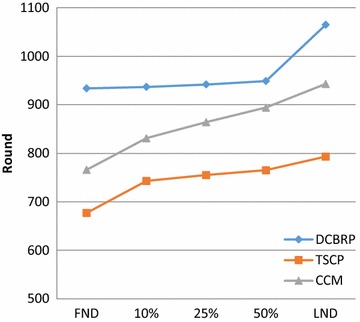
The network lifetime for DCBRP, TSCP and CCM

According to Fig. 8, the DCBRP has successfully extended the network lifetime, since all protocols started with the same energy level. The routing behaviour will directly affect the network lifetime because all mechanisms work together to reduce the energy consumption in the First-order Radio Model. This model has several important parameters, one of which is the distance in the BCM, CHS, and NHC mechanisms.

The primary finding from the FND is the ability of the routing protocol to extend the lifetimes of all nodes. Evidently, when the first node dies, the network loses one of its routing options, and this will lead to upper and lower nodes expending more energy for data delivery. However, the DCBRP prevents early node death, which is the main job of the third mechanism in the DCBRP (i.e., NHC). The NHC mechanism avoids using the weak nodes in the main chain and therefore performs only the sensing task. The CCM and TSCP rely on the sequence method for selecting the next-hop connection node.

### Energy consumption

Almost all evaluation strategies in WSNs include some form of energy metric (Mamun 2012). There are three energy metrics that are commonly used by the research community to investigate the energy efficiency of the DCBRP and compare it with other existing protocols. These metrics are discussed below.

- Total sensor-node energy consumption per round for all sensor nodes: This is considered an important metric for calculating the overall energy dissipation of all sensor nodes per round during the network lifetime, as shown in Eq. 20.
  20 $$E_{consu. in\;round\;r} = \mathop \sum \limits_{i = 1}^{number\;of\;nodes} E_{consu. in\;node\;i}$$
- Average energy consumption by the nodes over all rounds: This is used to study how the reduced energy consumption can prolong the network lifetime and is expressed in Eq. 21.
  21 $$E_{Av, Ene.consu} = \mathop \sum \limits_{firstround}^{lastround} E_{cons.allnodes} /N_{totalno.round}$$
- Average energy consumption by CHs in the network: This means that the average energy is consumed by the network CHs nodes in all rounds. This phenomenon is represented through Eq. 22.
  22 $$E_{Ave\;E\;consu\;by\;CH} = \mathop \sum \limits_{firstround}^{lastround} E_{ene.cons.byCH} /N_{totalNo.round}$$

**Fig. 9:**
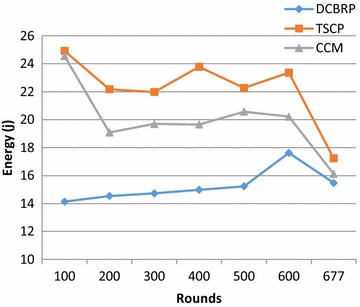
Energy consumption of DCBRP, TCSP and CCM

Figure 9 explains the energy consumption by all nodes within the DCBRP, TSCP and CCM. In this Figure, the DCBRP shows stable and smooth behaviours from early rounds until round number 600 in the specification of the CHS. The CHS mechanism selects the CHs based on the abilities of the nodes. It selects the same node until the comparative factor CH_Sfactor_ selects another node that is nearer than the previous CH. For this reason, the DCBRP network seems to be more stable compared to the CCM and TSCP. Hence, it does not expend extra energy to deliver data from other nodes and therefore keeps its energy for sensing purposes only for as long as possible.

**Fig. 10:**
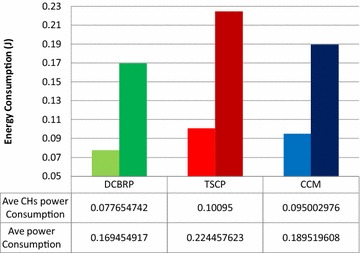
Average energy consumption for all nodes and for CHs nodes only

Although Fig. 10 presents the average energy consumption per round, it clearly shows that the DCBRP outperforms both the TSCP and CCM in terms of average energy consumption in every round during the network lifetime. That means that the DCBRP successfully reduces the energy depletion. The BCM builds the backbone chains and the CHS mechanism selects the chain heads depending on the CHS_factor_.

The DCBRP has compatible mechanisms working together to reduce delay and energy consumption. Therefore, when the nodes in the DCBRP expend approximately 0.17 J in each round, they provide a good indicator that the energy can be saved for the next rounds. However, the TSCP suffers from higher energy dissipation than the CCM because of the CH node chain connection.

Finally, average energy consumption for CHs is very critical issue because the DCBRP has more CHs in the network than CCM and TSCP, which have only one in their networks. Therefore, the energy consumption of CHs introduces a difficult challenge to the comparison. The DCBRP outperforms both protocols in terms of average CH energy consumption even though it has three CHs. The CHS mechanism, which assigns the same number of CHs and clusters in the network, divides the network over the CHs to provide higher energy savings. The CCM and TSCP place heavy loads on one main head, which may be in a different position than the BS, without considering the distance factor.

The sum of the average energy consumption for the DCBRP is 0.077 J, while it is 0.1 for the TSCP and 0.95 for the CCM. The DCBRP delivers the entire network’s data to the BS with balanced energy consumption among the CH nodes. In other words, the energy consumption by the main head in the CCM and TSCP is divided over the number of CHs in the DCBRP, since it has more than one CH connecting with the BS.

### Delay * Energy

This metric was suggested by Lindsey et al. (2001) and has been widely used by the research community for Chain-based routing in WSNs to combine the importance of energy consumption with the delay. This interesting metric can be calculated using Eq. 23.

23 $$Energy * delay = E_{total\;Econs. in\;round\;r} * D_{delay\;to\;dilever\;all\;data}$$

**Fig. 11:**
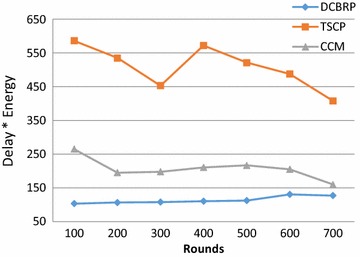
Delay * Energy metric for DCBRP, TSCP and CCM

Figure 11 shows that the DCBRP significantly outperformed the other protocols in terms of the Delay * Energy metric.

The DCBRP achieves good performance with respect to end-to-end delay and energy consumption by applying its mechanisms, i.e., BCM, CHS, and NHC. Consequently, the Delay * Energy factor will double when the multiplication operation is applied between two small numbers. Most importantly, it also tries to prolong the network lifetime.

The NHC mechanism also adds more stability to the DCBRP depending on the energy and distance of the node. The NHC mechanism considers all nodes in the network. The weak nodes are eliminated from the main chain without increasing the number of hops during packet transmission.

## Conclusion

This paper presents the DCBRP and focuses on the Chain-Head Selection mechanism (CHS). The CHS mechanism is responsible for selecting the chain-head node in each cluster in the network. This selection depends on the abilities of the nodes. In other words, the CHS_factor_ measures the ability of a node to deliver the network data with minimum energy consumption from its remaining energy. The node that has the smallest value of CHS_factor_ will be selected by the BS to become a CH for this round. The energy consumption is an important metric for the performance evaluation of the WSN. It is affected by the behaviour of the CHS mechanism and thus it is compared with the CCM and TSCP. The results show that the DCBRP outperforms the CCM and TSCP in terms of both node energy consumption and CH energy consumption. Furthermore, this superiority is a result of the behaviour of the CHS mechanism, in which the CHS depends on the CH_Sfactor_ and several CHs. The CCM depends on one main head and the sequential CH connection in cluster method, and the TSCP has one main head with a sequential CH connection in the chain concept. In summary, the DCBRP is superior to the CCM and TSCP with respect to the network lifetime metric, which is considered the main metric for measuring the node lifetimes in the WSNs. The network life is extended, which is the main objective of all WSN protocols.

## Future work

This research offers numerous potential new research trends for routing protocols in WSNs, such as:

- Applying the CHS mechanism in heterogeneous nodes to prolong the network lifetime should be investigated in the future,
- Applying the CHS mechanism with mobile BS to make energy balancing according to nodes distance with the BS may also be considered in the future, and
- Applying the CHS mechanism in random nodes deployment applications. This will be very useful for developing efficient protocol for random nodes’ deployment.

Furthermore, the DCBRP can be used in other sensor areas, such as the Internet of Things (IoT) (Gubbi et al. 2013) and/or used in cross layer protocol with MAC layer. Hence, the DCBRP can be considered as a promising protocol to be a base for any future work in the WSN because of its flexible mechanisms and Equations.
